# Items

**Published:** 1904-02

**Authors:** 


### ITEMS.

BATTLE & COMPANY, of Saint Louis, are issuing a series of bacteriological and blood charts, which should prove of interest to physicians. In March, 1904, pamphlet No. 1 of the series will be issued, which will contain 12 illustrations of the intestinal parasites.

A MEDICAL press exhibit is proposed to be given at the Saint Louis World's Fair. Space has been secured for that purpose in the palace of liberal arts by Dr. Charles Wood Fassett, editor of the Saint Joseph (Mo.) *Medical Herald,* who solicits the coöperation of editors and publishers of medical journals. Dr. Fasset should be addressed with promptitude on this subject.

MESSRS. FAIRCHILD BROTHERS & FOSTER, of New York, entertained their employes at luncheon December 24, 1903. The guests numbered about 140 persons and were assembled on the fourth floor of their large establishment.

Mr. B. T. Fairchild welcomed the guests to the "Christmas party," as it was called, and Mr. E. W. Dusenberry responded, after which Messrs. S. W. Fairchild and M. G. Foster also spoke. There was music during and after the luncheon, which began at 12.30, and at three o'clock the annual presents in gold were distributed among the employes, the remainder of the day being declared a holiday.

MESSRS. VICTOR KOECHL & COMPANY, of New York, have recently sent out a chart relating to acute poisoning, giving descriptive classification and varieties, with special symptoms, chemical and physiological antidotes, fatal doses, simple tests and remarks on treatment. It will be found a useful reminder to hang on the physician's office wall.

